# Th1 and Th17 Responses to *Helicobacter pylori* in Bangladeshi Infants, Children and Adults

**DOI:** 10.1371/journal.pone.0093943

**Published:** 2014-04-08

**Authors:** Taufiqur R. Bhuiyan, M M. Towhidul Islam, Taher Uddin, Mohiul I. Chowdhury, Anders Janzon, Jenni Adamsson, Samuel B. Lundin, Firdausi Qadri, Anna Lundgren

**Affiliations:** 1 International Centre for Diarrhoeal Disease Research, Bangladesh (icddr,b), Dhaka, Bangladesh; 2 Department of Microbiology and Immunology, Institute of Biomedicine, University of Gothenburg, Gothenburg, Sweden; University Medical Center Freiburg, Germany

## Abstract

Both Th1 and Th17 cells are important components of the immune response to *Helicobacter pylori* (Hp) in adults, but less is known about T cell responses to Hp during early childhood, when the infection is often acquired. We investigated Th1 and Th17 type responses to Hp in adults, children and infants in Bangladesh, where Hp is highly endemic. IL-17 and IFN-γ mRNA levels in gastric biopsies from Hp-infected Bangladeshi adults were analyzed and compared to levels in infected and uninfected Swedish controls. Since biopsies could not be collected from infants and children, cytokine responses in Bangladeshi infants (6–12 months), children (3–5 years) and adults (>19 years) were instead compared by stimulating peripheral blood mononuclear cells (PBMCs) with a Hp membrane preparation (MP) and analyzing culture supernatants by ELISA and cytometric bead array. We found significantly higher expression of IL-17 and IFN-γ mRNA in gastric mucosa of Hp-infected Bangladeshi and Swedish adults compared to uninfected Swedish controls. PBMCs from all age groups produced IL-17 and IFN-γ after MP stimulation, but little Th2 cytokines. IL-17 and IFN-γ were primarily produced by CD4^+^ T cells, since CD4^+^ T cell depleted PBMCs produced reduced amounts of these cytokines. Infant cells produced significantly more IL-17, but similar levels of IFN-γ, compared to adult cells after MP stimulation. In contrast, polyclonal stimulation induced lower levels IL-17 and IFN-γ in infant compared to adult PBMCs and CD4^+^ T cells. The strong IL-17 production in infants after MP stimulation was paralleled by significantly higher production of the IL-17 promoting cytokine IL-1β from infant compared to adult PBMCs and monocytes. In conclusion, these results show that T cells can produce high levels of IL-17 and IFN-γ in response to Hp from an early age and indicate a potential role for IL-1β in promoting Th17 responses to Hp during infancy.

## Introduction

*Helicobacter pylori* infects the gastric mucosa and causes gastritis in the majority of infected individuals. During acute *H. pylori* infection, neutrophils and macrophages accumulate in the mucosa as a result of early interactions between the bacteria, the epithelium and innate immune cells leading to production of increased levels of proinflammatory cytokines and chemokines, including IL-1β, TNF-α and IL-8 [1]–[3]. During later stages of the infection, T and B cells are also recruited to the mucosa and the chronic inflammation is characterized by the presence of both mononuclear and polymorphonuclear cells [2], [3]. Evidence suggests that the chronic inflammation is dependent on T cells, since *H. pylori* infection causes little inflammation in T cell deficient mice [4]. The T cell response to *H. pylori* is dominated by CD4^+^ helper T cells [2], [3], [5]. Many studies demonstrate that these cells produce IFN-γ and thus that the response is of a Th1 type [2], [5]–[7]. However, more recently, the importance of IL-17 producing Th17 T cells for mucosal immunity has become increasingly clear [8] and recent studies suggest that these cells also play an important role during *H. pylori* infection [2], [9]–[13].

Th17 cells produce several different cytokines, including the signature cytokine IL-17A (IL-17), which induces expression of chemokines that are neutrophil chemoattractants, including IL-8, from epithelial cells and fibroblasts [8], [14]. IL-17 producing cells have been shown to be important for protection against a number of different bacterial and fungal infections [8], [14]. IL-17 producing T cells may also contribute to *H. pylori* induced inflammation, since increased levels of both IL-17 mRNA and protein have been found in *H. pylori* infected compared to uninfected human mucosa [9]–[11]. Furthermore, the levels of IL-17 correlate with the levels of IL-8 as well as with the numbers of neutrophils infiltrating the mucosa [11], supporting that Th17 cells may be important components of active chronic gastritis in human *H. pylori* infection. However, the relative importance of Th1 versus Th17 responses for mucosal inflammation and protection remains unclear.

*H. pylori* infection is primarily acquired during childhood and in low and middle income countries, the age of acquisition is often very low [15]. We have recently shown that 50–60% of children in Bangladesh are infected already by the age of 2 years [16]. Some evidence suggests that *H. pylori* infection during early childhood can be transient in nature [15], [16]. Little is currently known about immune responses to *H. pylori* during early childhood, including factors that may promote spontaneous clearance of the infection. Studies in older children (about 10 years of age) indicate that increased levels of IFN-γ and IL-17 are found in infected compared to uninfected gastric mucosa also in children, but that the levels of inflammation may be lower in children compared to adults [17]–[20]. CD4^+^ T cells from children have been shown to have a reduced capacity to produce IFN-γ compared to adult T cells [21]; however, Th17 responses to *H. pylori* at different ages remain to be fully characterized. Increased knowledge about the ability of T cells from different age groups to produce cytokines implicated in protection against *H. pylori* may help the identification of vaccine regimens that have a potential to protect even young children and infants.

In this study, we analyzed T cell responses to *H. pylori* in infants, young children as well as adults from Bangladesh, where *H. pylori* infection is normally acquired during the first years of life, to determine if both Th1 and Th17 cells can respond to *H. pylori* antigens and may contribute to the immune response against this infection during childhood as well as adult life. We also characterize the cytokine profile of T cells from different age groups in response to polyclonal stimulation, including the general capacity of T cells from infants and children to produce IL-17.

## Materials and Methods

### Participants and sample collection

For analysis of cytokine responses to *H. pylori* antigens in circulating cells, 18 adults (19–32 years), 10 children (3–5 years) and 20 infants (6–12 months) were recruited to the study (Table 1) from the Mirpur area, 10 miles west of Dhaka city, Bangladesh. We chose the Mirpur site for our studies since it is representative of middle to low-income community, where we have experience in carrying out a large number of field and laboratory based studies. The participants did not have any symptoms of the infection and did not have any illnesses during the preceding three weeks before participation. Heparinized venous blood was collected once from each participant and transported directly to the laboratory. Stool samples were collected and stored at -70°C until tested for *H. pylori* infection using a stool antigen test.

**Table 1:** Demographic characteristics of participants recruited to the study.

Participants	n	Age^a^	Hp^+^/Hp^−b^	Females/Males
**Bangladesh (PBMC studies)**				
Infants	20	11.5 months (6–12)	12/8	10/10
Children	10	4.5 years (3–5)	5/5	6/4
Adults	18	27 years (19–32)	13/5	5/13
**Bangladesh (mucosal studies)**				
Adults	12	27 years (20–52)	11/1	0/12
**Sweden (mucosal studies)**				
Adults	19	55 years (27–82)	9/10	11/8

a Medians (range).

b Hp+; *H. pylori* infected, Hp-; *H. pylori* uninfected.

For analysis of mRNA levels in gastric biopsy material, 12 additional participants were recruited from the Mirpur area in Dhaka and 19 participants at the Sahlgrenska University Hospital in Sweden (Table 1). These participants did not have any gastric or duodenal ulcers, intestinal metaplasia or atrophy upon gastroscopy. Biopsies were collected from the gastric mucosa by gastroduodenal endoscopy and samples were stored in RNALater at −70°C until mRNA analysis.

The study was approved by the Ethical Committee of icddr,b as well as the Ethical Committee for Human Research in the Gothenburg region in Sweden and all participants or parents of the children and infants provided written informed consent before participation.

### Membrane protein preparation

A membrane protein preparation (MP) was prepared from strain Hel 305 by sonication followed by differential centrifugation [22] and used for both T cell stimulation and for serological ELISA assays. The Hel 305 strain was originally isolated from a Swedish duodenal ulcer patient and carries an intact Cag pathogenicity island and expresses VacA s1/m1 This genotype is also common in Bangladeshi *H. pylori* strains [23]. Western blot analysis showed that the Hel 305 MP contains many different proteins, including urease, NAP, HpaA and flagellin, as well as LPS (<50% wt/wt).

### Diagnosis of *H. pylori* infection

To determine the *H. pylori* status in Bangladeshi infants, children and adults, fresh stool samples were collected from all participants and stored at −70°C until tested for *H. pylori* using a monoclonal antibody based stool antigen kit (Amplified IDEIA Hp StAR, Dakocytomation) according to the manufacturer's instructions. The *H. pylori* status of the Swedish participants was determined by culture of *H. pylori* bacteria on blood Columbia iso agar plates and the infection status was confirmed by serology, using Hel 305 MP as coating antigen in the ELISA tests [24]. We have previously compared the stool antigen test both to bacterial culture [25] and serology [16], [26] and found good agreement between results from the different tests. We have also demonstrated that the use of MP from the Swedish *H. pylori* strain Hel 305 or MP from Bangladeshi strains in the serology ELISA test give comparable results [16], [25].

### Cytokine gene expression analysis

RNALater-stabilized human tissue specimens were thawed in 600 μl buffer RLT (Qiagen) with 1% β-mercaptoethanol and homogenized by bead-milling in a TissueLyser II (Qiagen) as described [27] followed by isolation of RNA using Qiagen's RNeasy Mini kit according to the manufacturer's instructions. The isolated RNA was checked for integrity by agarose gel electrophoresis and the concentration was measured using spectrophotometry (NanoDrop Technologies). Six hundred ng of RNA was then used for cDNA using the Omniscript Reverse Transcription kit (Qiagen) as described elsewhere [28]. Relative expression of IL-17 and IFN-γ mRNA:s was subsequently determined with quantitative real-time PCR gene expression assays from Applied Biosystems using HPRT as an internal reference gene. The assays were performed as previously described [28] and gene expression changes data were analyzed using the ΔΔCt method [29], calculating fold change of each gene, normalized to the reference gene and relative to an external calibrator sample consisting of antral biopsy cDNA from an individual outside the study groups.

### Cell isolation and stimulation

Peripheral blood mononuclear cells (PBMCs) were isolated by gradient centrifugation on Ficoll-Isopaque (Pharmacia). CD14^+^ monocytes were isolated from PBMCs using MACS beads (Miltenyi Biotec) and CD4^+^ T cells were isolated or depleted from PBMCs using Dynabeads (Invitrogen), according to the instructions provided by the manufacturers. All cells were incubated in 96-well U bottomed tissue culture plate (Nunc) in DMEM F12 medium (Invitrogen) with 5% human serum and 1% gentamicin at 37°C in 5% CO_2._ DMEM F12 was used in these studies, since we have found that higher levels of IL-17A can be detected both after short and long term T cell stimulation in this medium, compared to other media such as RPMI, while IFN-γ levels are less affected by the culture medium used (A. Lundgren, unpublished data).

PBMCs and PBMCs depleted of CD4^+^ T cells (1.5×10^5^ cells/well, 200 μl/well) as well as CD14^+^ monocytes (1×10^5^ cells/well, 200 μl/well) were stimulated with Hel 305 MP (1 μg/ml). We have previously shown that T cells respond in a comparable way to stimulation with MP, *H. pylori* lysate and whole *H. pylori* bacteria [30]. Since Hel 305 MP has been used extensively in our previous studies of T cell [5], [7], [30] and antibody [16], [24]–[26] responses to *H. pylori*, we chose to use this antigen for stimulation of T cells also in this study. The MP concentration was titrated in initial experiments and 1 μg/ml was found to give rise to strongest and most consistent responses. PBMCs and CD4^+^ depleted PBMCs were also stimulated with phytohemagglutinin (PHA, Remel, 1 μg/ml).

Purified CD4^+^ T cells (2.5×10^4^ cells/well, 200 μl/well) were stimulated with anti-CD3/anti-CD28 coated expansion beads (Invitrogen) at a cell:bead ratio of 1∶1. Supernatants (150 μl per well) were collected after 48 hours of MP stimulation for analysis of IL-1β, IL-6, IL-12 and IL-23 and after 48 hours of bead stimulation, or after 5 days of MP or PHA stimulation, for analysis of IFN-γ, IL-17A, IL-4, IL-5, IL-13, IL-10 and TNF-α. The time points for sample collection were chosen based on previous studies showing that IL-17 and IFN-γ responses have different kinetics after antigen stimulation with IL-17 responses peaking later than IFN-γ responses but with high levels of both cytokines detected in supernatants after 5 days of culture (A. Lundgren, unpublished data). The samples were immediately frozen at −70°C until assayed for cytokines.

### Cytokine analysis

To analyse the levels of different cytokines in cell culture supernatants, ELISA (IL-17A, IFN-γ, IL-1β, IL-6, IL-12p70 and IL-23; eBioScience; IL-13; R&D) and cytometric bead array (CBA; IL-2, IL-4, IL-5, IL-10, TNF-α, Becton Dickinson (BD)) assays were performed according to the instructions provided by the manufacturers.

### Flow cytometric analysis

For analysis of the frequencies of different cell subsets among the PBMCs and for analysis of the purity of CD4 PBMCs and isolated CD4^+^ and CD14^+^ cells, cells were stained with combinations of the following antibodies: anti-CD14 FITC, anti-CD4 PerCP, anti-CD19 PE, anti-CD3 APC, anti-CD45RO PE and anti-CD8 FITC (BD). Cells were analyzed using FACSCalibur, equipped with blue and red lasers (BD) and the flow cytometric data was analyzed with FlowJo software (Tree Star Inc.). Isolated CD4^+^ cells contained >95% CD3^+^CD4^+^ cells. Isolated CD14^+^ T cells contained >95% CD14^+^ cells. The frequency of CD4^+^ in PBMCs after depletion of CD4^+^ cells was <5%.

### Data analysis

Data were analyzed using Graphpad Prism version 5.0 and SigmaStat 3.1 programs (SPSS Systat software, Inc). The Mann-Whitney U-test and the Wilcoxon matched pairs test were used for statistical analysis. *P* values <0.05 were considered to be statistically significant.

## Results

### Cytokine expression in gastric mucosa

**Figure 1:**
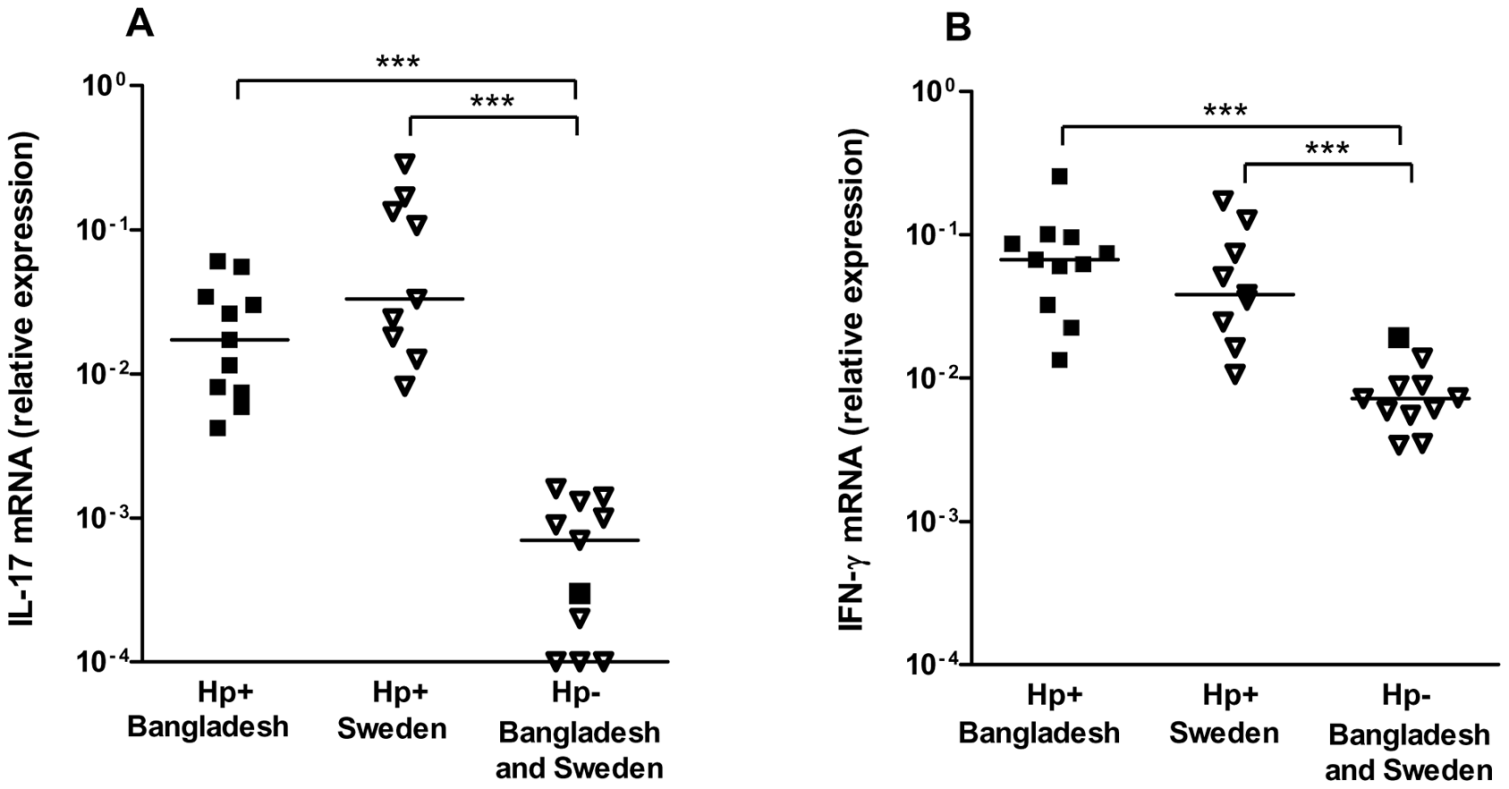
Expression of IL-17 and IFN-γ in gastric mucosa. Levels of IL-17 (A) and IFN-γ (B) mRNA were analyzed in gastric biopsy material collected from *H. pylori* infected (Hp+, filled symbols) and uninfected (Hp-, open symbols) Bangladeshi (squared symbols) and Swedish (triangular symbols) adults. The gene expression data is indicated as fold change normalized to an internal reference gene (HPRT) and relative to the calibrator sample. Horizontal lines represent median values (***P<0.001).

To determine if the mucosal immune response in Bangladeshi individuals is characterized by expression of both IL-17 and IFN-γ, as previously reported from studies from other parts of the world [9]–[11], [17], [20], the expression of IL-17 and IFN-γ mRNA was analyzed in gastric biopsies collected from Bangladeshi adults (Figure 1). Since it is difficult to get access to biopsy material from uninfected Bangladeshi participants as a consequence of the high prevalence of *H. pylori* infection in this population, we mainly used biopsies from uninfected and infected Swedes for comparison. We found that both IL-17 and IFN-γ was expressed in the gastric mucosa of *H. pylori* infected Bangladeshi individuals and that the levels were comparable to those detected in gastric mucosa of infected Swedish participants. The expression of both cytokines was significantly higher in infected participants from both Sweden and Bangladesh, compared to in uninfected Swedish controls. Similar low levels of mRNA for both cytokines were also detected in material obtained from one uninfected Bangladeshi individual (Figure 1). These results support that the mucosal immune response to *H. pylori* is characterized by expression of both IL-17 and IFN-γ in Bangladeshi as well as Swedish subjects.

### T cell cytokine responses to *H. pylori* MP in PBMCs from different age groups

**Figure 2:**
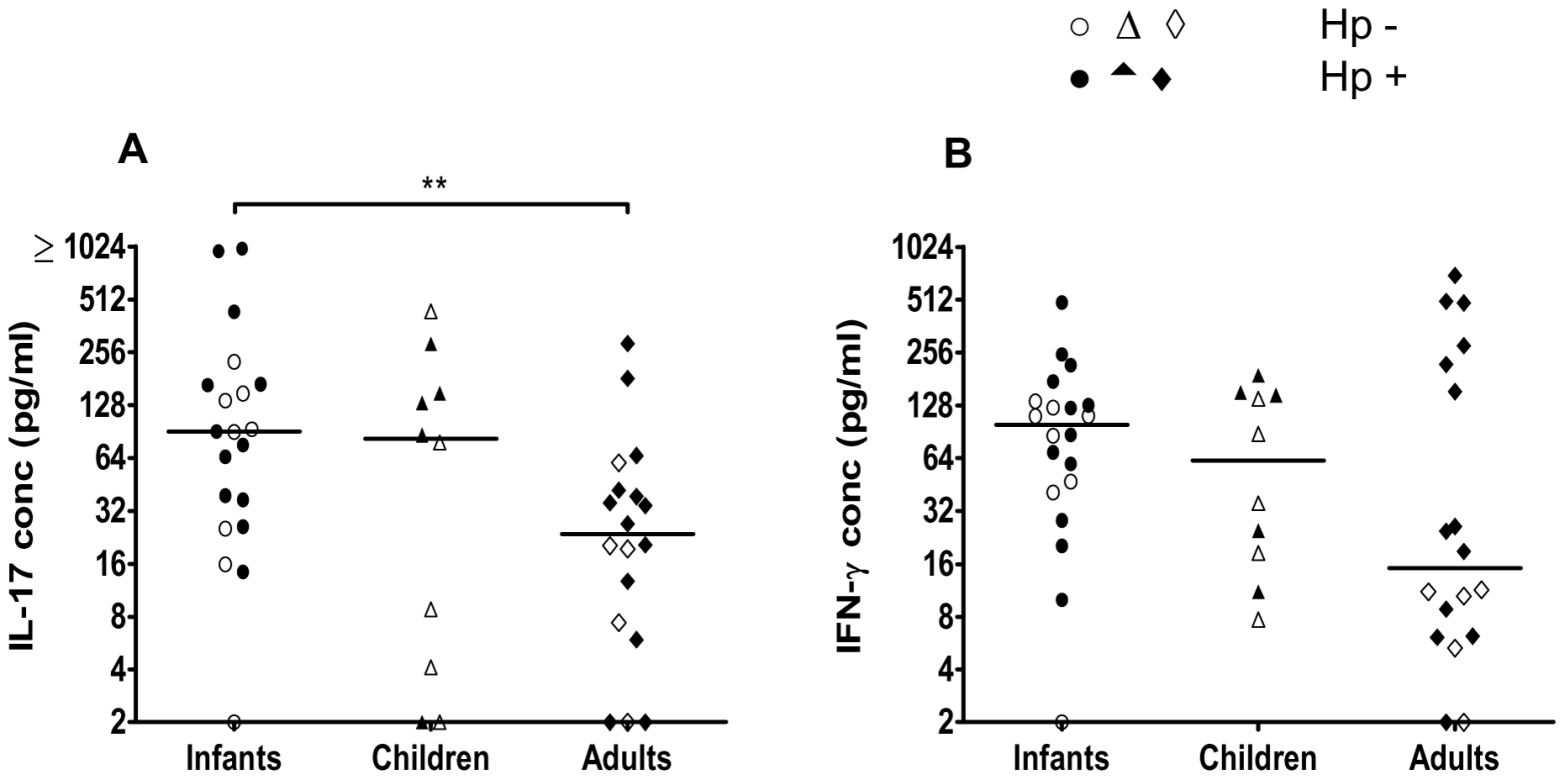
IL-17 and IFN-γ responses to *H. pylori* antigens in PBMCs. IL-17 (A) and IFN-γ (B) production from PBMCs isolated from Bangladeshi infants (n = 20), children (n = 10) and adults (n = 18) was analyzed after stimulation with *H. pylori* MP. Filled symbols represent *H. pylori* infected participants (Hp+) and open symbols *H. pylori* uninfected participants (Hp-). Responses to medium alone have been subtracted from the values shown. Horizontal lines represent median values (**P<0.01).

Since biopsies could not be collected from infants and children, the cytokine profiles of T cells responding to *H. pylori* in different age groups were instead analyzed by stimulating PBMCs from both *H. pylori* infected and uninfected infants (6–12 months old), children (3–5 years) and adults (>19 years) with *H. pylori* MP and measuring the presence of different cytokines in culture supernatants by ELISA and CBA. We found that PBMCs from all age groups produced IL-17 and IFN-γ upon stimulation with *H. pylori* antigens (Figure 2). The levels of IL-17 produced in response to *H. pylori* MP were significantly higher in supernatants collected from infants compared to adults (Figure 2A). There was also a tendency for higher IL-17 production in PBMCs from children compared to adults, but this difference was not statistically significant. The production of IFN-γ was comparable in the different age groups. PBMCs from infants and adults also produced comparable levels of IL-10, but little IL-13, IL-5, IL-4, IL-2 or TNF-α in response to *H. pylori* MP (Table 2). Analysis of samples from a subset of the children participating in the study indicated that cells from children and infants produce comparable levels of these cytokines (data not shown).

**Table 2:** Levels of different cytokines in culture supernatants after stimulation of PBMCs with *H. pylori* MP or PHA.

	MP		PHA	
	Infants	Adults	Infants	Adults
**IL-13**	10 (0–90)	5 (0–85)	112 (32–732)	307 (46–1700)
**IL-5**	10*** (0–113)	0 (0–1)	69 (0–175)	88 (13–178)
**IL-4**	0 (0–6)	0 (0–4)	0 (0–37)	0 (0–18)
**IL-10**	178 (56–592)	278 (38–510)	93 (0–2305)	206 (63–390)
**TNF-α**	18 (0–364)	14 (0–417)	21 (0–327)	93 (0–468)
**IL-2**	10 (0–74)	2 (0–32)	0 (0–58)	0 (0–13)

Cytokines were measured by ELISA (IL-13, adults; n = 8 and infants; n = 9) and CBA (IL-5, IL-4, IL-10, TNF-α, IL-2; adults n = 13 and infants; n = 17). Cytokine levels (pg/ml) are expressed as medians (range) after subtraction of responses to medium alone. Lower numbers of samples could be analyzed for IL-13 than the other cytokines, since the IL-13 ELISA analysis required larger sample volumes than the CBA analysis.

***P<0.001; infants versus adults.

To determine if the cytokine responses reflect the infection status of the participants, we also compared the cytokine responses in *H. pylori* infected (Figure 2, filled symbols) and uninfected (open symbols) participants. There were no significant differences in responses in infected and uninfected subjects in any of the age groups, although a subgroup of infected adults tended to produce high levels of IFN-γ. However, when the whole group of infected adults was compared to the uninfected adults, the difference between the groups was not statistically significant (*P*>0.05).

### Origin of the cytokines produced in responses to *H. pylori* MP

**Figure 3:**
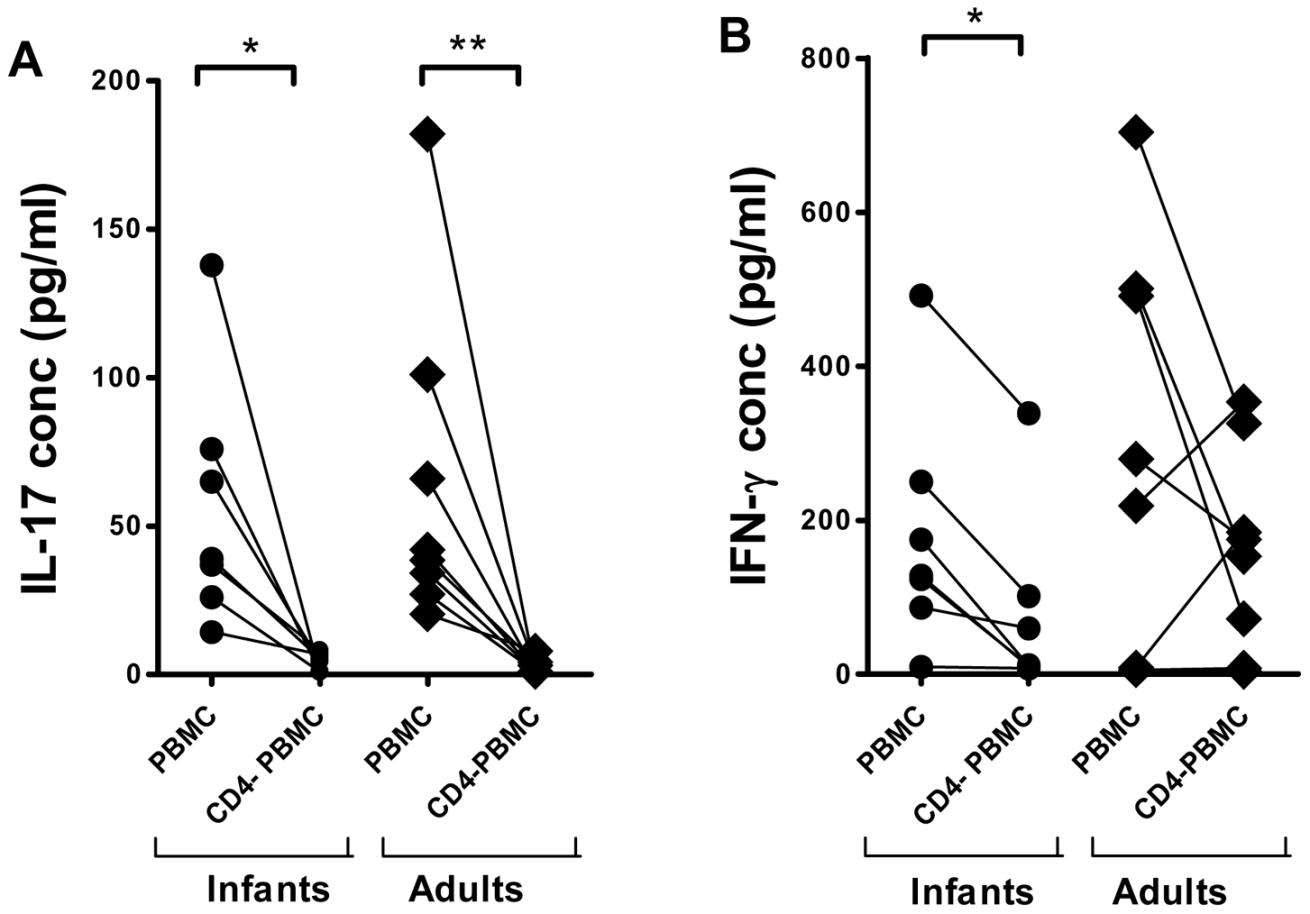
Effects of CD4^+^ T cell depletion on IL-17 and IFN-γ responses to *H. pylori* antigens in PBMCs. Production of IL-17 (A) and IFN-γ (B) was analyzed in PBMC and PBMC depleted of CD4^+^ T cells (CD4^−^ PBMC) after stimulation with *H. pylori* MP in infants (n = 7) and adults (n = 8). Responses to medium alone have been subtracted from the values shown (*P<0.05, **P<0.01).

To determine if CD4^+^ T cells were responsible for the observed cytokine responses, we depleted CD4^+^ T cells from the PBMCs and stimulated both PBMCs and CD4^−^ PBMCs with *H. pylori* MP. We found that depletion of CD4^+^ T cells abolished the production of IL-17 in cells from both infants and adults (Figure 3A). Depletion of CD4^+^ T cells also strongly reduced the production of IFN-γ in all infants and a majority of the adults, although responses in two adults increased after depletion. Depletion had variable effects on the levels of IL-10 in different individuals (data not shown).

To control for the possibility that the higher levels of IL-17 detected in culture supernatants from infants compared to adults could be a result of higher frequencies of CD4^+^ T cells in PBMCs isolated from infants, we analyzed the frequencies of CD4^+^ T cells among PBMCs isolated from infants and adults. CD3^+^CD4^+^ cells represented about 30% of all PBMCs in both infants and adults (Table 3). The proportion of memory (CD45RO^+^) cells among the CD4^+^ T cells was about fourfold higher in adults than in infants (*P*<0.001, Table 3). Similar frequencies of CD14^+^ monocytes (Table 2) and CD19^+^ B cells (data not shown) were also found in infants and adults.

**Table 3:** Frequencies of different cell types in PBMCs isolated from infants and adults.

	CD3^+^CD4^+a^	CD45RO^+b^	CD14^+c^
Infants	34 (22–48)	17 (12–34)***	5 (3–13)
Adults	32 (24–53)	63 (47–86)	11 (3–13)

a Frequency (%) of CD3^+^CD4^+^ cells among all cells.

b Frequency (%) of CD45RO^+^ cells among CD3^+^CD4^+^ cells

c Frequency (%) of CD14^+^ cells among all cells.

Values are expressed as medians (range). n = 10 for adults and n = 10 for infants.

^***^P <0.001; infants versus adults.

### Production of cytokines by antigen presenting cells that may influence T cell responses

**Figure 4:**
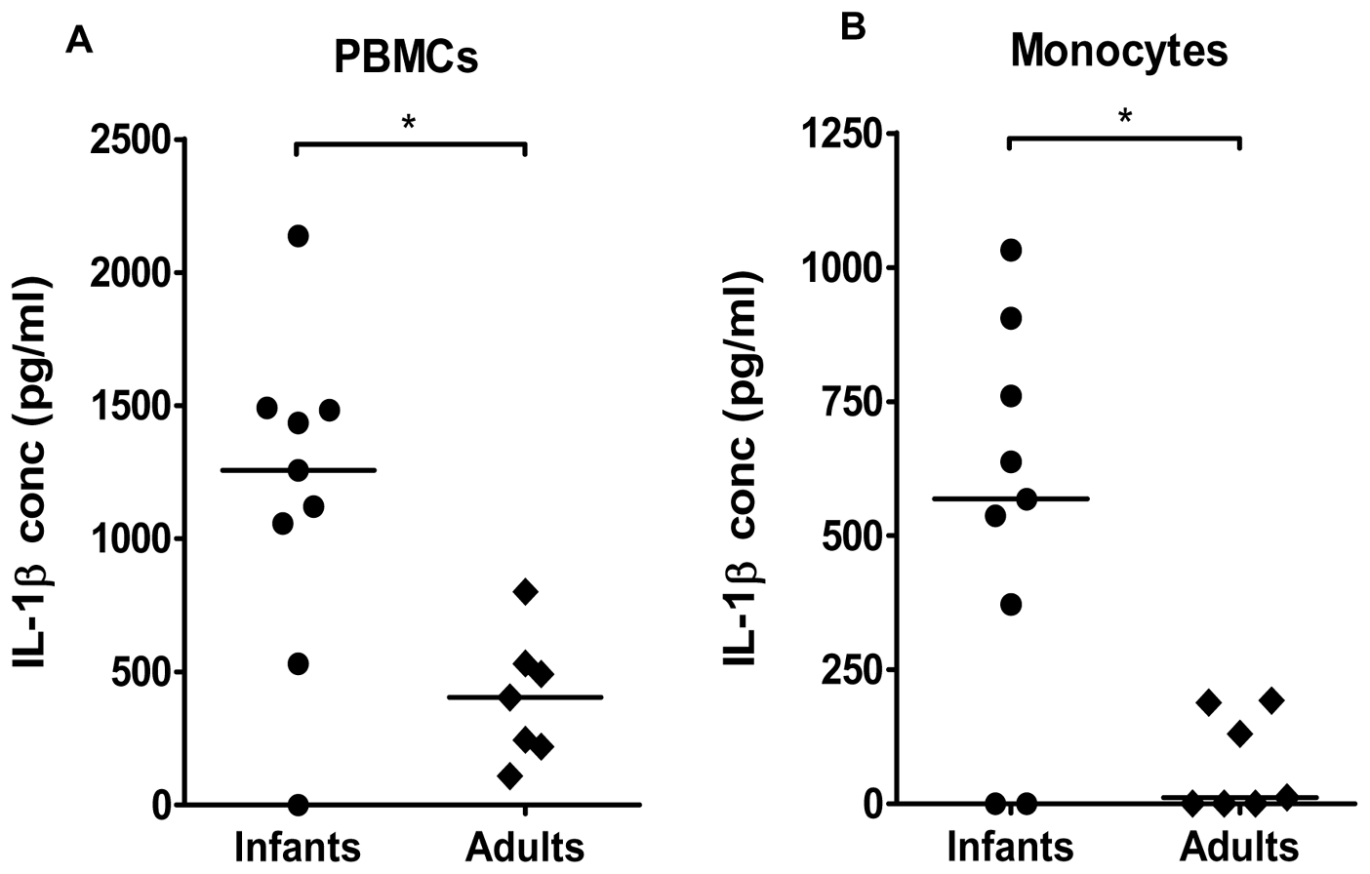
IL-1β production from PBMCs and monocytes in response to *H. pylori* antigens. IL-1β production from PBMCs (A) and CD14^+^ monocytes (B) isolated from *H. pylori* infected infants (n = 9) and adults (n = 7) was analysed after stimulation with *H. pylori* MP. Responses to medium alone have been subtracted from the values shown. Horizontal lines represent median values (*P<0.05).

We hypothesized that the relatively strong production of IL-17 observed in PBMCs from infants in response to MP stimulation may be a result of high production of Th17 promoting cytokines from antigen presenting cells (APCs) in this study group. To address this hypothesis, we analyzed the levels of the cytokines IL-1β, IL-6 and IL-23, which are known to induce, enhance and/or sustain IL-17 responses in human T cells [14], [31]. After two days of MP stimulation, PBMCs isolated from infants had produced significantly higher levels of IL-1β than PBMCs isolated from adults (Figure 4A). In contrast, PBMCs from the two study groups produced comparable levels of IL-6 and IL-23 (data not shown). The levels of IL-12, which support IFN-γ responses, were close to or below the detection limit (2 pg/ml) in most samples (data not shown).

Monocytes are potent producers of IL-1β [32]. To investigate if monocytes from infants are able to produce high levels of IL-1β, and thereby capable of supporting strong T cell production of IL-17, the cytokine production from isolated CD14^+^ monocytes from infants and adults was analyzed after stimulation with MP. We found that monocytes from infants produced significantly more IL-1β in response to MP stimulation, compared to monocytes from adults (Figure 4B). In contrast, levels of IL-6, IL-23 and IL-12 were comparable in monocyte cultures from infants and adults (data not shown).

### Cytokine responses after polyclonal stimulation

**Figure 5:**
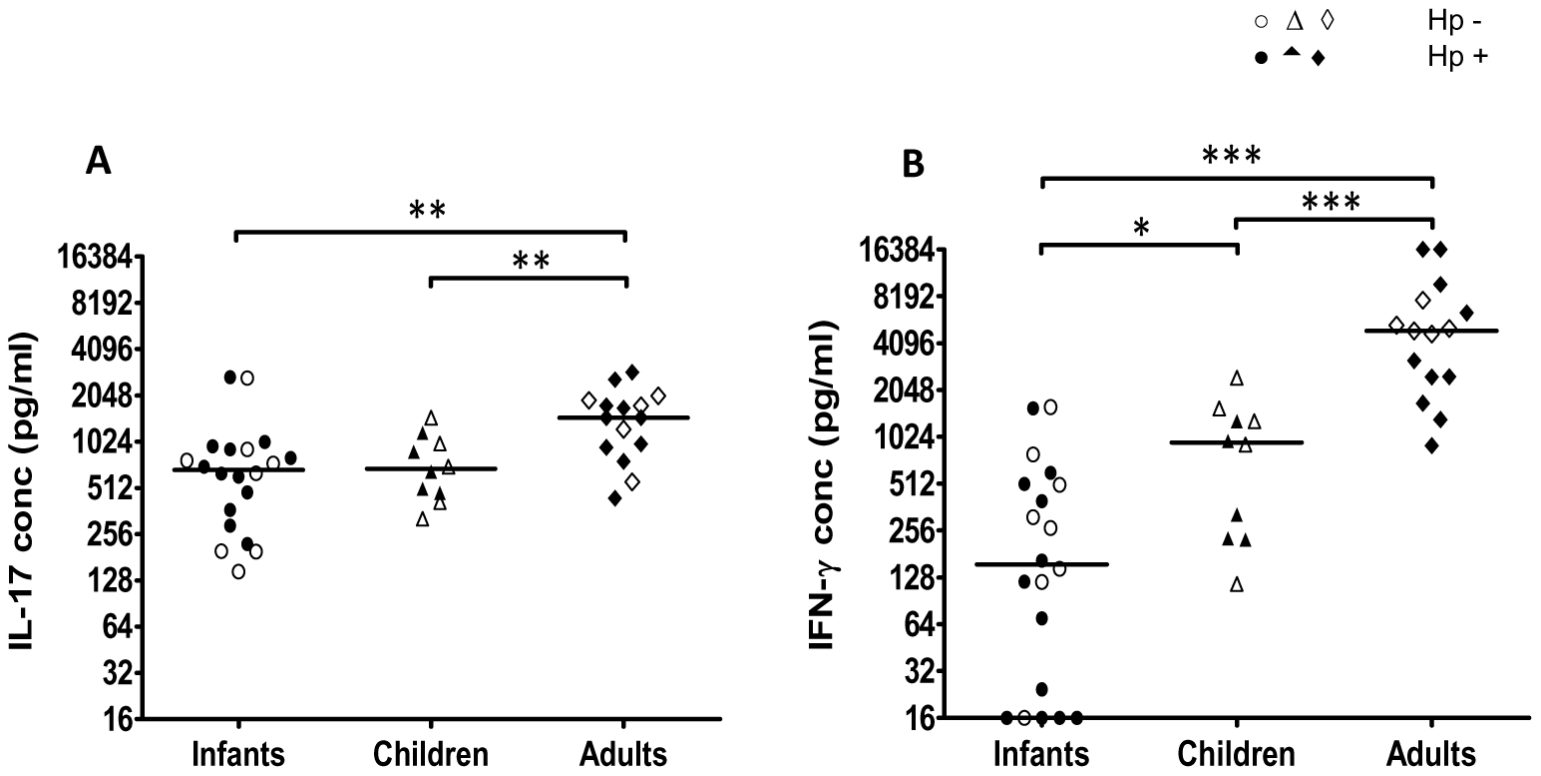
Polyclonal IL-17 and IFN-γ responses in PBMCs after PHA stimulation. IL-17 (A) and IFN-γ (B) production from PBMCs isolated from infants (n = 20), children (n = 10) and adults (n = 15) was analyzed after PHA stimulation. Filled symbols represent *H. pylori* infected participants (Hp+) and open symbols represent *H. pylori* uninfected participants (Hp-). Responses to medium alone have been subtracted from the values shown. Horizontal lines represent median values (*P<0.05, **P<0.01***P<0.001).

To determine whether the observed MP induced responses reflect the general ability of T cells to produce cytokines, the cytokine secretion in response to the mitogen PHA was also analyzed. The pattern of responses to PHA differed from that observed after MP stimulation, since PBMCs from adults produced significantly higher levels of both IL-17 and IFN-γ than cells from infants (Figure 5). The IFN-γ responses were particularly age dependent, with significantly higher response levels in children compared to infants (Figure 5B), whereas IL-17 responses to PHA were similar in these two age groups (Figure 5A). PHA stimulation also induced a relatively high and comparable production of IL-13 in infants and adults (Table 2). Relatively low IL-10, IL-4, IL-5, TNF-α and IL-2 responses were detected in response to PHA (Table 2). Analysis of samples from a subset of children suggested that cells from children and infants produce comparable levels of these cytokines after PHA stimulation (data not shown). *H. pylori* infected and uninfected individuals responded with comparable cytokine secretion to stimulation with PHA (Figure 4 and data not shown).

**Figure 6:**
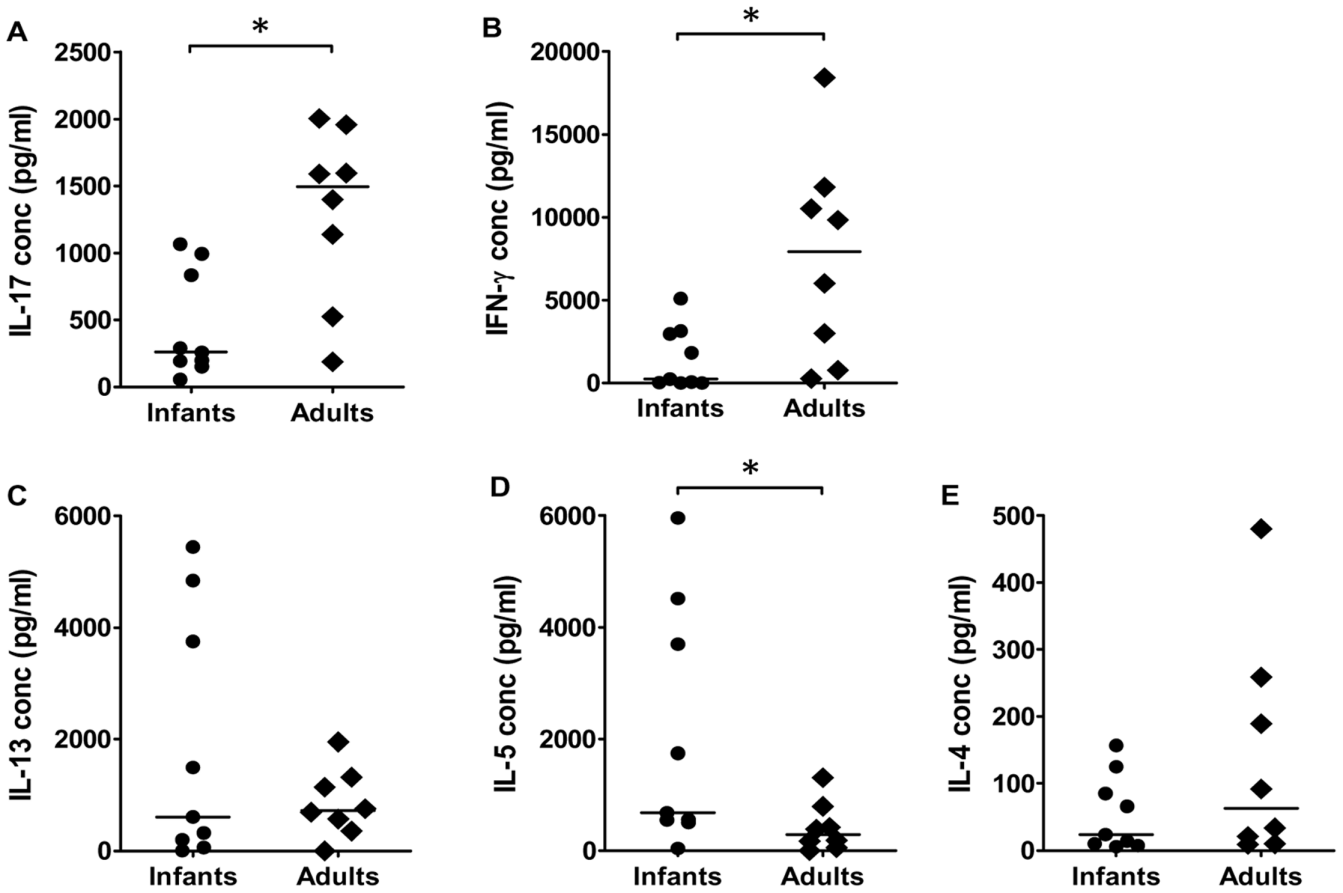
Polyclonal cytokine responses in CD4^+^ T cells after anti-CD3/CD28 stimulation. Production of IL-17 (A), IFN-γ (B), IL-13 (C), IL-5 (D) and IL-4 (E) by CD4^+^ T cells isolated from *H. pylori* infected infants (n = 9) and adults (n = 8) was analyzed after stimulation with beads coated with anti-CD3 and anti-CD28 antibodies. Horizontal lines represent median values (*P<0.05).

To analyze and compare the cytokine profiles of CD4^+^ T cells from infants and adults without the influence of cytokines and other signals delivered APCs and other cells present in the cultures, we stimulated purified CD4^+^ T cells with beads coated with anti-CD3 and anti-CD28 antibodies in the absence of any accessory cells. Similar to the responses observed after PHA stimulation of PBMCs, CD4^+^ T cells from adults produced significantly higher levels of IL-17 and IFN-γ compared to infants in response to bead stimulation (Figure 6). In contrast, significantly higher levels of IL-5 were observed in infants compared to adults (Figure 6 D). However, similar levels of IL-13 and IL-4 (Figure 6 C and E) as well as IL-10, IL-2 and TNF-α (data not shown) were produced by CD4^+^ T cells from infants and adults.

Taken together, these results demonstrate that the IL-17 and IFN-γ producing phenotype of T cells responding to *H. pylori* antigens is different from the polyclonal cytokine profile of CD4^+^ T cells.

## Discussion

IL-17 is currently believed to be an important cytokine for orchestrating the immune response to *H. pylori* [2], [12], but little is known about Th17 responses to this infection in young children and infants or in individuals in low and middle income countries. In this study, we demonstrate comparable expression of IL-17 and IFN-γ in *H. pylori* infected gastric mucosa from Bangladeshi and Swedish adults, which was significantly higher than the expression in uninfected controls. This extends our previous results, showing that the numbers of T and B cells as well as *H. pylori* specific IgA antibody levels are also comparable in adult gastric mucosa from Bangladeshi and Swedish volunteers [25]. Our results thus demonstrate that the mucosal immune response to the infection is characterized by expression of Th1 and Th17 type cytokines in both populations.

PBMCs from Bangladeshi infants, children and adults produced both IL-17 and IFN-γ in response to stimulation with *H. pylori* antigens. While PBMCs from infants produced the highest levels of IL-17, cells from children produced slightly less and the lowest amounts were produced by adult cells. In contrast, IFN-γ responses to *H. pylori* antigens were comparable in the different age groups. Depletion experiments showed that CD4^+^ T cells was the major source of IL-17 but that some production of IFN-γ remained after depletion of CD4^+^ T cells. This is consistent with earlier reports of IFN-γ secretion from both CD8^+^ T cells and NK cells in response to *H. pylori* antigens [33], [34]. In agreement with most previous studies [2], [5], little Th2 cytokines were produced in response to MP stimulation. Relatively large amounts of IL-10 were however detected, but this cytokine may be produced by CD4^+^ T cells as well as other cells, including monocytes, which may partly explain why CD4^+^ T cell depletion had variable effects on IL-10 production in different donors.

We investigated whether the strong IL-17 production in infant T cells may be related to secretion of IL-17 promoting cytokines from infant APCs. We found that infant PBMCs and monocytes produced significantly more IL-1β than cells from adults after stimulation with MP, while the production of IL-6 and IL-23 were comparable in the different groups. IL-1β can promote IL-17 production in both memory and naive T cells [31]. Recent studies also show that *in vitro* blockade of the IL-1 receptor leads to reduced IL-17 responses to *H. pylori* antigens [35]. We therefore find it likely that the strong IL-1β production in infant APCs may at least partly explain the high production of IL-17 in this age group. Infant APCs have previously been reported to produce comparable or lower levels of IL-1β than adult cells, but the responses may vary depending on the type of stimuli [36], [37]. The MP preparation contains many different proteins, including urease, flagellin as well as LPS. This preparation clearly provides a mix of signals that gives rise to prominent IL-1β production in infant APCs. *In vitro* neutralization of IL-1β was not performed in this study to test the role of this cytokine for promoting IL-17 responses due to the low number of cells that could be isolated from infants and children. We also believe that the most important effects of IL-1β on T cell polarization is likely to take place *in vivo* rather than *in vitro*.

Our demonstration of potent IL-1β production from infant cells is important, since this indicates that infant APCs may be able to strongly support Th17 type T cell responses to both infections and immunizations, if the right stimulatory signals are provided. We have recently shown that the adjuvant activity of the novel mucosal adjuvant double-mutant heat labile toxin (dmLT) may at least partly be dependent on IL-1β [38] and studies in mice suggest that this adjuvant can promote protective Th17 responses when administered in combination with *H. pylori* vaccines [39]. Further studies are clearly warranted to study IL-1β responses in infant APCs and how these may influence responses to different antigens, adjuvants and vaccines.

IFN-γ and IL-17, as well as the proinflammatory cytokines IL-1β, IL-23 and IL-6, are expressed in stomach mucosa from both children and adults [1], [9], [13], [17], [19], suggesting that production of IL-1β may indeed promote IL-17 production in the gastric mucosa. However, studies in children about 10 years of age suggest that children may have lower gastric inflammation with reduced neutrophil infiltration and lower expression of IL-17 than adults [18], [20]. To our knowledge this is the first description of IL-17 responses to *H. pylori* in very young children and infants. Our results suggest that cells from young children have the ability to produce more IL-17 and IL-1β than adult cells in response to *H. pylori* under the right stimulatory conditions. However, further studies are needed to determine if the strong IL-17 production detected in circulating T cells correlates with high IL-17 production in the mucosa of infants and young children, or if local responses may be partially suppressed by Treg, as indicated in older children [17], [18], [20]. We have previously shown that 50-60% of Bangladeshi children acquire *H. pylori* during the first two years of life [16]. About 10% of these children spontaneously eradicate the infection, which is associated with increased production of antibodies [26]. However, it is unclear whether these antibodies are directly involved in bacterial clearance or if they rather correlate with other types of protective responses. We speculate that IL-17 and/or IFN-γ producing CD4^+^ T cells may help to clear the infection in infants and children, but additional studies are needed to address this hypothesis and to determine why the infection is not cleared in all children.

Production of IL-17 and IFN-γ may contribute to protection against *H. pylori* via several different mechanisms. IL-17 can promote the recruitment of neutrophils [14], which may penetrate the gastric epithelium and kill *H. pylori* bacteria in the lumen [40]. IL-17 may also enhance production of antimicrobial peptides [14]. IFN-γ may contribute to the inflammatory process by enhancing production of chemokines. Studies using gene knockout mice to address the role of IL-17 in *H. pylori* colonization and associated inflammation have not led to conclusive results [12], [41]–[44] partly due to the fact that *H. pylori* infection leads to very mild inflammation in mice. On the other hand, immunization with *H. pylori* lysate antigens has been shown to induce strong IL-17 responses with local infiltration of neutrophils. Furthermore, IL-17 neutralization or neutrophil depletion inhibits vaccine induced reduction of *H. pylori* colonization [45]–[47], supporting that the IL-17-neutrophil axis may be an important protective mechanism against *H. pylori*.

Our findings of comparable IL-17 and IFN-γ responses in PBMCs from infected and uninfected subjects is likely to be at least partly explained by the high exposure to *H. pylori* in Bangladesh and the relatively high spontaneous clearance rate in children in this population [16], [26]. However, since circulating T cells from both infected and uninfected individuals in countries with lower prevalence of *H. pylori* also respond similarly to *H. pylori* antigens after both short and longer stimulation [7], [48], [49], we cannot rule out that the responses detected may also partially be a result of cross-reactivity with other bacteria expressing similar antigens. Due to the small amount of blood available, we could only test MP from one *H. pylori* strain. T cells may respond slightly differently to antigen preparations from other strains and this will be investigated in future studies.

Our analysis of the polyclonal responses of T cells confirms that CD4^+^ T cells from infants and children are capable of potent production of IL-17 and IFN-γ. However, the strongest polyclonal IL-17 and IFN-γ responses were found in adults. Since memory cells are the major source of IL-17 after both polyclonal and antigen stimulation [50], [51], this is likely to be explained by the higher frequency of memory T cells found among adult compared to infant PBMCs. Our findings of higher production of IL-5 in CD4^+^ T cells from infants compared to adults in response to anti-CD3/CD28 stimulation in the absence of APCs are in agreement with previous studies showing a general Th2 bias of T cells from young children [52]. We have recently described strong IL-17 and IFN-γ responses to *Streptococcus pneumoniae* in the same Bangladeshi population [51], providing further support that robust Th1 and Th17 type responses can develop despite this potential general Th2 skewing.

In conclusion, our results demonstrate that the mucosal immune response to *H. pylori* is characterized by expression of IL-17 and IFN-γ in Bangladeshi adults and that CD4^+^ T cells from adults, children and infants can respond to *H. pylori* antigens with production of both IL-17 and IFN-γ. The IL-17 production was particularly pronounced in cells isolated from infants, which was paralleled by high production of IL-1β, indicating a role of this cytokine in promoting Th17 type T cell responses to *H. pylori* in infants. Considering the growing evidence that Th17 type responses provide protection against *H. pylori* after immunization in mice, our results provide hope that a combination of the right antigens and adjuvant in a future *H. pylori* vaccine may induce protective immunity even in infants.
